# Macrophage activation syndrome, a rare complication of primary Sjögren’s syndrome: a case report

**DOI:** 10.1186/s13256-019-2252-z

**Published:** 2019-10-16

**Authors:** B. S. Kane, M. Niasse, A. Faye, N. D. Diack, B. Djiba, M. Dieng, M. Sow, A. C. Ndao, N. Diagne, S. Ndongo, A. Pouye

**Affiliations:** 1 Department of Internal Medicine, Cheikh Anta DIOP University, Dakar, Senegal; 2 Department of Rheumatology, Cheikh Anta DIOP University, Dakar, Senegal

**Keywords:** Macrophage activation syndrome, Sjögren’s syndrome, Africa south of the Sahara, Case report

## Abstract

**Background:**

The association of macrophage activation syndrome and primary Sjögren’s syndrome has been rarely reported in the literature. We report the first observation of this association in Africa, south of the Sahara, and we discuss the diagnosis and therapeutic challenge.

**Case presentation:**

A 26-year-old Mauritanian and Berber woman was followed for primary Sjögren’s syndrome. After a voluntary cessation of her usual background treatment, she was admitted to our department for an outbreak of her illness. A clinical examination revealed anemic syndrome, peripheral polyarthritis, coughing rales at both pulmonary bases, and fever at 39.5 °C. On biologic examination, there was bicytopenia with anemia at 5.70 g/dl, lymphopenia at 796/mm^3^, a biological inflammatory syndrome with a sedimentation rate at 137 mm in the first hour, C-reactive protein at 97 mg/l, hyperferritinemia at 1778 mg/l (9 normal value), and hypergammaglobulinemia at 20.7 g/l of polyclonal appearance. The triglycerides were 1.95 g/l (1.4 normal value) and the lactate dehydrogenase level was 491 IU/l (1.5 normal value). Cytological examination of a medullary puncture revealed an image of hemophagocytosis. An infectious screening was negative. Thoracic computed tomography showed non-specific interstitial lung disease. A diagnosis of macrophage activation syndrome complicating primary Sjögren’s syndrome was selected with a probability of 97.2%, according to H-Score. The evolution was favorable under a treatment including etoposide (VP-16).

**Conclusion:**

Macrophage activation syndrome is a rare entity, rarely reported during primary Sjögren’s syndrome. Its spontaneous evolution is invariably fatal. There is no consensus on therapeutic treatment. Etoposide is a therapeutic option especially in forms refractory to corticosteroid therapy.

## Background

Macrophage activation syndrome (MAS) is a phenomenon characterized by cytopenia, organ dysfunction, and coagulopathy associated with an inappropriate activation of macrophages [1]. It remains a rare pathology, with a guarded prognosis, characterized by clinical and biological signs that are not very specific and whose association helps invoke the diagnosis [2]. The syndrome may be primary and hereditary or secondary to a neoplastic, infectious, or autoimmune condition or to immunosuppressive therapies. The term MAS has been used mainly to designate secondary forms, including autoimmune diseases [3]. During these inflammatory conditions, it has mainly been described in association with Still’s disease and systemic lupus, and in exceptional cases related to primary Sjögren’s syndrome (SS) [4–6]. We report the first observation of this association in Sub-Saharan Africa and we discuss the diagnosis and therapeutic challenges.

## Case presentation

A 26-year-old Mauritanian and Berber woman was followed for 3 years for primary SS. This diagnosis was made on the basis of the following factors: dry eye syndrome, chronic non-erosive peripheral arthritis, positive Schirmer’s test, anti-SSA > 8 IU antibodies, and chronic lymphocyte grade IV sialadenitis (based on Chisholm and Mason classification). The remainder of immunological assessment showed a titer of antinuclear antibodies at 1:80, and autoantibodies to double-stranded deoxyribonucleic acid (anti-dsDNA antibodies), rheumatoid factor, and anti-citrullinated peptide antibodies (ACPA) were negative.

This autoimmune disease was treated with prednisone (5 mg daily), hydroxychloroquine (400 mg daily), and methotrexate (15 mg weekly). Three months after stopping her disease-modifying antirheumatic drugs (DMARD), she was hospitalized for acute fever and inflammatory arthralgia.

At admission, she had a temperature of 39.5 °C, heart rate of 120 beats/minute, blood pressure of 110/80 mmHg, and breathing rate of 22 cycles/minute. A musculoskeletal examination showed synovitis of her wrists and knees. A pleuropulmonary examination revealed asymmetric crackling rales at the pulmonary bases. The activity of the disease was evaluated at 35 on the European League Against Rheumatism Sjögren’s Syndrome Disease Activity Index (ESSDAI). The rest of the clinical examination was within normal limits; notably, there was no hepatosplenomegaly or lymphadenopathy.

The laboratory tests showed a bicytopenia with a biological inflammatory syndrome and hyperferritinemia. We summarized the laboratory data of our patient during her last visit before the loss of follow-up and during the hospitalization in Table 1. A bone marrow aspiration showed hyperplasia of the granular lineage with plasmacytosis and images of hemophagocytosis. At this stage the diagnosis of MAS was retained with an H-Score of 219 points and a diagnostic probability of 93–96% (Table 2).

**Table 1:** Laboratory results

Last visit before loss of follow-up	Hospitalization
WBCs, 6100/mm^3^	WBCs, 4670/mm^3^
Hemoglobin, 11.9 g/dl	with neutrophils 3736/mm^3^
Platelets, 350,000/mm^3^	Bicytopenia with lymphopenia at 796/mm^3^ and hemoglobin at 5.7 g/dl
ESR, 86 mm	Platelets, 245,000/mm^3^
CRP, 93 mg/l	ESR, 137 mm
ALT, 6 UI/l	CRP, 97 mg/l
AST, 14 UI/l	Fibrinogen, 2 g/l
	Blood protein electrophoresis, polyclonal hypergammaglobulinemia at 20.7 g/l
	Hyperferritinemia, 1778 ng/ml (9 N)
	LDH, 491 IU/l (1.5 N)
	Triglycerides, 195 mg/dl
	AST, 35 UI/l
	ALT, 31UI/l
	Serum creatinine, 4.92 mg/l
	Proteinuria (g/24 hours), 0.6 g/24 hours
	Serum calcium, 81 mg/l
	Serum phosphorus, 30 mg/l
	Serum vitamin B12, 253 pg/ml

*ALT* alanine transferase, *AST* aspartate transferase, *CRP* C-reactive protein, *ESR* erythrocyte sedimentation rate, *LDH* lactate dehydrogenase, *N* normal value, *WBCs* white blood cells

**Table 2:** H-Score for reactive hemophagocytic syndrome in our patient

Criteria	Score
Know underlying immunosuppression (that is, HIV positive or receiving long-term immunosuppressive therapy, such as glucocorticoids, cyclosporine, and azathioprine	Yes (+ 18 points)
Temperature (°C)	39.5 °C (+ 49 points)
Organomegaly	No (0)
Number of cytopenia (defined as hemoglobin ≤ 9.2 g/dl and/or WBC ≤ 5000/mm^3^ and/or platelets ≤ 110,000/mm^3^)	2 lineages (+ 24 points)
Ferritin (ng/ml)	1778 (0)
Triglyceride (mg/dl)	195 (+ 44)
Fibrinogen (g/l)	2 (30)
AST (U/L)	35 (+ 19 points)
Hemophagocytosis features on bone marrow aspirate	Yes (+ 35 points)
Total	219 points

Probability of hemophagocytic syndrome in our patient is 93–96%. Optimal cut-off 169 points. *AST* aspartate transferase, *WBC* white blood cell, *HIV* human immunodeficiency virus

Screening for infections: the *Plasmodium* thick blood test, blood cultures, cytobacteriological examination of urine, cytobacteriological examination of sputum, acid-fast bacillus (AFB) search (for *Mycobacterium tuberculosis*), Epstein–Barr virus (EBV) polymerase chain reaction (PCR), and human immunodeficiency virus (HIV) serology were all negative. Standard radiography revealed interstitial syndrome at the pulmonary bases of the thorax. Thoracic computed tomography showed non-specific interstitial lung disease. The diagnosis of primary SS complicated by interstitial lung disease and MAS was retained.

**Fig. 1:**
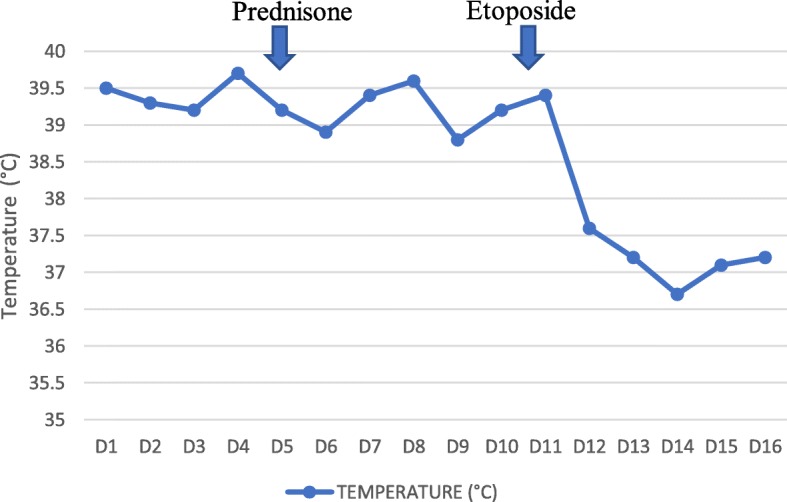
Curve of temperature

Initial treatment was based on an increase in corticosteroid therapy to 1 mg/kg per day with blood transfusion. The outcome of her clinical condition, after 1 week of treatment, was marked by persistence of an intermittent fever with peaks at 39–40 °C. Etoposide treatment was initiated at a rate of 150 mg/m^2^ (200 mg in a single intravenous injection). Her clinical course was marked by a clear improvement in the symptomatology, with stable apyrexia, a C-reactive control protein of 13.7 mg/l, and a hemoglobin level of 8.2 g/dl obtained after the first 24 hours (Fig. 1). On discharge, she was switched to Imurel (azathioprine; 100 mg/day) and hydroxychloroquine (400 mg/day) combined with corticosteroid therapy. A follow-up 2 months later, with a good adherence and tolerance of the treatment (patient self-assessment), showed a complete regression of the cytopenia, a negative C-reactive protein, and serum ferritin at 224 μg/l.

## Discussion

This study describes a case of MAS complicating primary SS.

In Sub-Saharan Africa, MAS has been the subject of limited publications [7–10]. Its pathophysiology is incompletely elucidated. Recent studies have implicated a defect in the cytotoxicity of T and natural killer (NK) lymphocytes following a stimulus, leading to a massive release of cytokines responsible for macrophage activation with hemophagocytosis and clinico-biological manifestations [3].

The diagnosis of MAS is a real challenge [11]. From our observation, it included fever, cytopenia, hyperferritinemia, hypertriglyceridemia, and the demonstration of hemophagocytosis in bone marrow aspirates. Our patient’s H-Score was rated at 219 points with a diagnostic probability of 93–96%. This score was recently developed to assess the diagnostic probability of secondary hemophagocytic lymphohistiocytosis (HL) in adults [12, 13].

For an etiological approach, it is necessary to distinguish primary HL occurring especially at the pediatric age, from “reactive” HL. These “reactive” forms are secondary to infections, cancers, immunosuppressive therapeutics, and autoimmune disease [2, 11]. The term, MAS, was used to designate forms secondary to autoimmune and auto-inflammatory disease [11, 14]. Therefore, MAS can help reveal or follow the diagnosis of the underlying condition [15]. In our observation, it occurred during the clinical course of primary SS.

In relation to inflammatory diseases, MAS has been more frequently reported in juvenile idiopathic arthritis in its systemic form, but also in systemic lupus erythematosus, adult-onset Still’s disease, Kawasaki disease, dermatomyositis, mixed connective tissue disease, systemic sclerosis, and primary SS [11]. The MAS–SS association has been rarely reported. In a systematic review analyzing 117 publications about MAS in systemic diseases, and including 421 patients, only 3 cases were associated with SS [16]. This case is, to the best of our knowledge, the first report of this association in Africa.

HL occurring during autoimmune diseases can be distinguished as two entities: a form associated with an active infection notably favored by immunosuppressive treatments and a form specifically associated with the severity and the activity of the autoimmune disease [6]. In our patient, the severe progression of SS, evidenced by the disease activity and occurrence of interstitial lung disease, could be considered a precipitating factor of the MAS.

However, an underlying infectious origin, particularly EBV, and other agents such as tuberculosis, visceral leishmaniasis, malaria, salmonellosis, parvovirus B19, or dengue fever should always be excluded [17]. For our patient, the EBV-PCR was negative; however, the PCR for other viral agents such as cytomegalovirus (CMV) and parvovirus B19 was not carried out because of our economic limitations. The screening for the most common infectious diseases, such as tuberculosis, in tropical areas was negative.

There is no consensus on the therapeutic approach for MAS in autoimmune diseases [14, 16]. In the absence of infection, some authors have proposed the use of high doses of corticosteroids as a first-line treatment [3]. In refractory forms, other molecules such as cyclosporin A, etoposide, or intravenous immunoglobulins could be considered second-line options [3]. For our patient, the clinical course quickly became favorable on administration of etoposide. This molecule treats HL by selectively eliminating pathologic, activated T cells lymphocytes with an efficient suppression of inflammatory cytokine production [18]. It is an effective and emergency treatment of MAS associated with SS.

## Conclusion

MAS is a rare entity, which is rarely reported during primary SS. For treatment, an underlying infection must be excluded. Its spontaneous clinical course invariably proves fatal. There is no consensus on therapeutic treatment; however, etoposide is one option, especially in forms refractory to corticosteroid therapy.

## Data Availability

Not applicable.
